# Use, perceptions, and barriers to supraglottic airway devices in Brazil: a national cross-sectional survey with global implications

**DOI:** 10.1016/j.bjane.2026.844760

**Published:** 2026-04-27

**Authors:** Jayme M. dos Santos Neto, Flávia J. de Barros, Victor M. Lemos, Clístenes Crístian de Carvalho

**Affiliations:** a Hospital das Clínicas da Universidade Federal de Pernambuco (HC-UFPE), Recife, PE, Brazil; b Universidade Federal de Campina Grande (UFCG), Campina Grande, PB, Brazil; c Guy's and St. Thomas' NHS Foundation Trust, Department of Anaesthesia and Perioperative Medicine, London, UK

**Keywords:** Airway management, Anesthesiology, Continuing medical education, Laryngeal masks, Patient safety, Surveys and questionnaires

## Abstract

**Background:**

Supraglottic Airway Devices (SADs) are essential in airway management. However, little is known about their use, perceptions, and barriers in large, resource-variable healthcare systems such as Brazil. We aimed to assess current practice patterns, training exposure, and barriers to SADs use among Brazilian anesthesiologists and trainees.

**Methods:**

A nationwide, web-based survey that addressed demographics, training exposure, device availability, frequency of use, perceived safety and efficacy, barriers to use, and knowledge of SADs generations. Descriptive and inferential statistics were used to analyze regional and experiential patterns.

**Results:**

A total of 432 completed responses were analyzed. Most respondents were fully trained anesthesiologists (75.5%) from all five Brazilian regions. Although SADs were widely perceived as safe (76.9%) and capable of providing adequate ventilation (74.3%), only 35% reported using them at least once per week, and fewer than 20% felt confident distinguishing between first and second-generation devices. Binomial logistic regression revealed that greater training exposure during residency was strongly associated with more frequent current use (OR = 7.3; 95% CI 3.8‒14.3, p < 0.001). The most common barrier was lack of operator experience (81.5%), followed by limited availability of devices (28.9%). Use varied across regions (p  < 0.001), with 82% of anesthesiologists in the South reporting weekly use compared to only 15.4% in the North. Respondents were more likely to use SADs in limb surgeries, while use is limited in gastrointestinal and thoracic procedures.

**Conclusions:**

Despite favorable perceptions of safety and efficacy, SADs remain underused in Brazil, with marked regional variation and limited familiarity with device generations. Training exposure emerged as a strong predictor of current use, underscoring the importance of structured education and hands-on experience. Addressing gaps in training and improving device availability may help standardize practice and enhance the use of SADs across the country.

Institutional Research Board approval n° 7.214.701; CAAE: 84060624.6.0000.8807.

## Introduction

Airway management is a cornerstone of anesthetic practice and a critical determinant of patient safety.^1^ Over recent decades, Supraglottic Airway Devices (SADs) have become established as reliable, versatile tools in both routine and emergency settings.^2^ Their ease of insertion, favorable safety profile, and applicability in both spontaneous and controlled ventilation have made them integral to modern anesthesia practice. Second-generation SADs are increasingly favored due to improved oropharyngeal sealing, integrated gastric channels, and reduced risk of aspiration.^2^, ^3^, ^4^, ^5^

A growing body of evidence from large observational studies and randomized trials supports the efficacy and safety of second-generation SADs across diverse clinical contexts, including complex surgeries and high-risk populations.^4^^,^^6^^,^^7^ For instance, a prospective international cohort study reported a 98.6% success rate with the i-gel Plus, with minimal complications and high ease-of-use ratings.^6^ Additionally, real-world studies have shown that transitioning from first to second-generation SADs improves airway safety in routine care.^7^ A recent systematic review and meta-analysis further demonstrated that, in adults undergoing abdominopelvic surgery, second-generation SADs reduced major airway complications and significantly improved postoperative recovery outcomes compared to tracheal intubation.^4^

Despite this compelling evidence, significant variability in SADs usage persists across countries and institutions.^1^ Disparities in training, device access, and risk perception continue to shape practice patterns. Brazil, home to one of the largest and most diverse healthcare systems globally, represents a critical yet underexplored setting in this context.

As a country of continental dimensions, Brazil exhibits substantial regional disparities in healthcare infrastructure, resource allocation, and anesthesiology training. While regions such as the South and Southeast mirror high-income systems in terms of practice and access, others, particularly in the North, face challenges more typical of low-resource environments.^8^ Thus, national data from Brazil can serve as a proxy for the broader challenges encountered in resource-variable global contexts. Yet, little is known about how Brazilian anesthesiologists currently use SADs, perceive their safety and efficacy, or experience barriers to their adoption.

Accordingly, this study aimed to evaluate current practice patterns, perceptions of safety and efficacy, and barriers to the use of supraglottic airway devices among anesthesiologists in Brazil.

## Methods

This cross-sectional study was based on an anonymous electronic questionnaire distributed to eligible participants. The research adhered to the ethical principles outlined in the Declaration of Helsinki and complied with Resolution 466/12 of the Brazilian National Health Council. Ethical approval was granted by the study’s hospital Research Ethics Committee (approval #7.214.701; CAAE: 84060624.6.0000.8807). This manuscript conforms to established reporting standards and adheres to the Checklist for Reporting of Survey Studies (CROSS).^9^, ^10^, ^11^

A link to the survey, accompanied by a brief cover letter outlining the study’s objectives, was created for national distribution and subsequently deactivated after the study period. Before accessing the questionnaire, participants viewed an online information sheet describing the study objectives, procedures, voluntary nature of participation, right to withdraw at any time, and contact details for the investigators. Electronic informed consent was obtained by selecting an “I agree to participate” option. Respondents who did not provide consent could not proceed to the survey.

The final version of the questionnaire comprised 26 questions across 11 sections: General Information; Experience in Anesthesiology; Professional Practice; Experience with Supraglottic Devices; Use of Supraglottic Devices in Elective General Anesthesia; Perceived Safety of Supraglottic Airway Devices; Adequacy of Ventilation with Supraglottic Airway Devices; Barriers to Supraglottic Airway Devices; Knowledge of Device Generation; Scenarios for Use; and Continuing Education (Appendix S1). The survey was designed to assess clinical use, perceived safety and efficacy, confidence and training, availability, and barriers to the adoption of supraglottic airway devices across a range of clinical scenarios. The primary outcomes were defined and established *a priori* at initiation of the study design.

Initially consisting of 33 items, the questionnaire was refined over four iterative rounds of review by the research team. A pretest was conducted with four anesthesiologists to evaluate clarity and technical functionality. Responses from the pretest phase were excluded from the final analysis. After minor adjustments, the finalized version of the survey was locked and not modified thereafter.

The link was distributed twice by the Brazilian Society of Anesthesiology (SBA) via institutional email to all registered members and was additionally shared by the research team through social media platforms (WhatsApp® and Instagram®) to enhance dissemination. Eligible participants included board-certified anesthesiologists and anesthesiology residents with a minimum of three months of specialty training. The survey remained open from November 27, 2024, to January 2, 2025.

Study data were collected and managed using REDCap electronic data capture tools hosted at the study hospital’s university.^12^^,^^13^ REDCap (Research Electronic Data Capture), developed by Vanderbilt University (Nashville, TN, USA), is a secure, web-based software platform designed to support data capture for research studies, providing: 1) An intuitive interface for validated data capture; 2) Audit trails for tracking data manipulation and export procedures; 3) Automated export procedures for seamless data downloads to common statistical packages; and 4) Procedures for data integration and interoperability with external sources. The data were downloaded as a comma-separated values (.csv) file, and only fully completed questionnaires were included in the analysis.

Sample size estimation was performed using the ‘samplesize4surveys’^14^ package in R software (R Foundation for Statistical Computing, Vienna, Austria), adopting a conservative approach. Considering the total membership of the SBA as the population (n = 13,265), we assumed a 95% confidence level, a 3% margin of error, and a conservative estimated prevalence of 10% of anesthesiologists using a SAD at least once per week. Under these assumptions, the required sample size was calculated to be 374. Anticipating a low response rate of approximately 5% via email and social media distribution, we estimated that outreach to at least 7480 members would be necessary. To maximize participation and enhance representativeness, the survey was sent to all 13,265 SBA members.

### Statistical analysis

All statistical analyses were performed using JAMOVI (version 2.0, MacOS X) and R (version 4.4.2). Descriptive statistics were used to summarize respondent characteristics and survey responses, with results presented as means ± standard deviation for continuous variables, and as absolute frequencies and percentages for categorical variables. For ordinal and non-normally distributed variables, nonparametric tests were applied, including the Kruskal-Wallis rank sum test for regional comparisons and Spearman’s rank correlation to assess the association between training exposure and current use of SADs. A two-sided p-value of < 0.05 was considered statistically significant.

A binomial logistic regression was conducted considering the responses of fully trained anesthesiologists only. The independent variables of interest were frequency of use during anesthesiology specialization, self-reported experience with SADs, knowledge of device generation, age (45 < vs. ≥ 45) and professional workplace (public or private hospital). Bivariate analyses (Chi-Square or Fisher's exact test) were employed to determine the association between each independent variable of interest and the outcome (use of the device). Bivariate models with p < 0.30 and theoretically relevant variables in the context of the study were selected for the multiple model. The model fit was checked using deviance and AIC (Akaike Information Criterion) with results presented as odds ratio values, 95% confidence intervals, and statistical significance. For all analyses, a p-value < 0.05 was considered.

## Results

A total of 587 responses were collected through REDCap® following survey distribution to all 13,265 members of the Brazilian Society of Anesthesiology, resulting in an overall response rate of approximately 4.4%. After excluding incomplete submissions and entries without signed consent, 432 fully completed questionnaires were included in the final analysis, yielding a usable response rate of 3.3%. Respondents who identified as students were automatically excluded via survey logic.

Participants were predominantly fully trained anesthesiologists (75.5%) from all five Brazilian regions, though the majority were based in the Northeast (53.9%). Most respondents worked in general hospitals (91.9%) and had substantial clinical exposure, with over 80% involved in more than 150 general anesthesia procedures annually (Table 1).

**Table 1:** Demographic and professional characteristics of survey respondents (n = 432).

Variable		n/Average	%/DP
Age (years)		39.6	10.9
Gender			
	Female	181	41.9
	Male	249	57.6
	Not declared	2	0.5
Level of training			
	Resident/Specializing in Anesthesiology	106	24.5
	Anesthesiologist	326	75.5
Region of operation			
	North	12	2.8
	Northeast	233	53.9
	Midwest	25	5.8
	Southeast	112	25.9
	South	50	11.6
Workplace			
	General Hospital	398	91.9
	Specialized hospital	155	35.8
	Day hospital	117	27.0
Profile of the work institution			
	Public	253	58.4
	Private	238	55.0
	Mixed	138	31.9
Number of surgical procedures with general anesthesia per year			
	0–50 (up to 1-per week)	24	5.6
	51–150 (1–3 per week)	58	13.4
	> 150 (more than 3-per week)	350	81.0

SD, Standard Deviation.

Most participants reported at least moderate experience with SADs: 39.6% indicated “moderate experience”, 33.8% “extensive experience”, and 15.5% considered themselves experts (Table 2). Frequent clinical use was reported by 35.4% (at least once a week) and 25.0% (at least once a month). SADs were reportedly always available in 70.6% of workplaces and partially available in 28.9%.

**Table 2:** Experience with, use and availability of Supraglottic Airway Devices (SADs).

Variable		n	%
Experience with SADs			
	No experience	4	0.9
	Minimal experience	44	10.2
	Moderate experience	171	39.6
	Extensive experience	146	33.8
	Expert level	67	15.5
Use of SADs in elective procedures			
	Once a year or less	57	13.2
	Two to three times a year	51	11.8
	Four to six times a year	63	14.6
	Once a month or more	108	25.0
	Once a week or more	153	35.4
Availability of SADs in the performance services			
	Never available	0	0
	Partially available	125	28.9
	Only available for emergencies	2	0.5
	Always available	305	70.6

**Figure 1:**
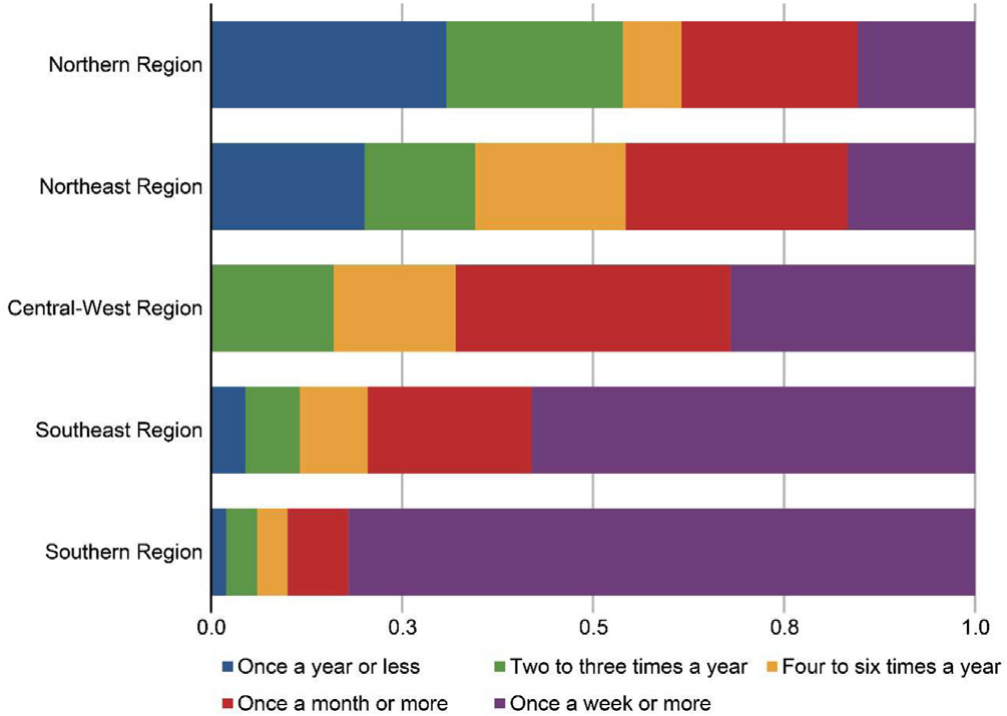
Regional variability in the frequency of Supraglottic Airway Devices (SADs) use in Brazil. This horizontal stacked bar chart displays the distribution of reported frequency of SADs use in elective general anesthesia across Brazil’s five geographic regions. The Y-axis lists the regions, while the X-axis shows the percentage of respondents within each region. Colored segments represent frequency categories, ranging from “Once a year or less” to “Once a week or more”, as indicated in the legend. Respondents from the South most commonly reported weekly use (82%), whereas in the North, only 15.4% reported using SADs weekly and 30.8% reported use once a year or less, highlighting pronounced regional disparities in clinical practice.

Usage patterns varied substantially by region. In the South, 82% of respondents reported using SADs at least once per week, while in the North ‒ where overall use was markedly lower ‒ only 15.4% reported weekly use and 30.8% reported use once a year or less (Fig. 1). A statistically significant difference across regions was observed (Kruskal-Wallis test, χ²(4) = 107.49, p < 0.001).

**Figure 2:**
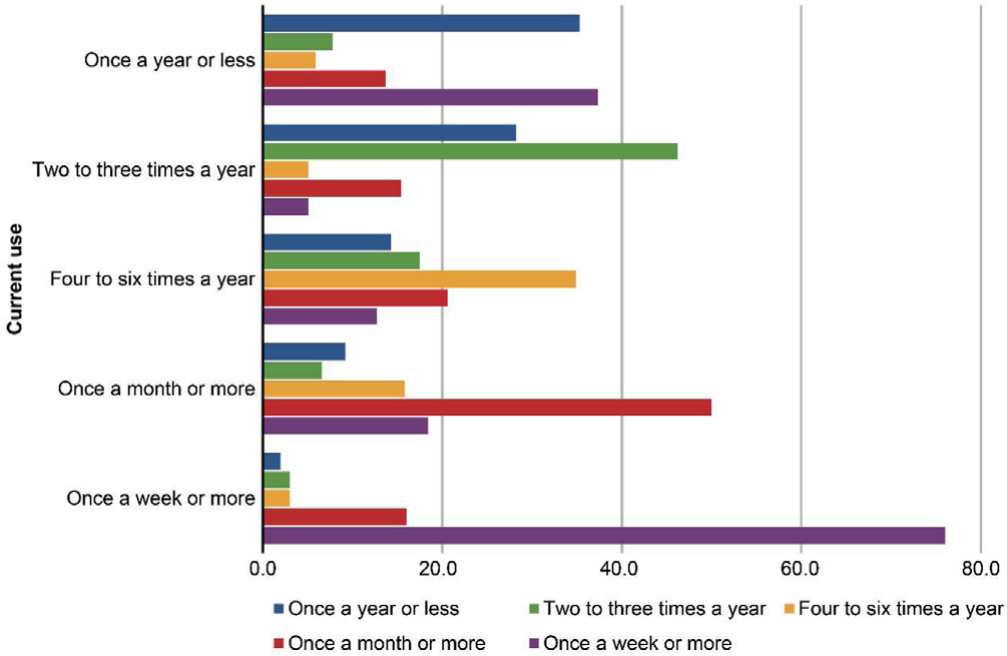
Association between training exposure during residency and current use of Supraglottic Airway Devices (SADs). This bar chart illustrates the relationship between the frequency of SADs use during anesthesiology residency (represented by colored bars) and current use in elective general anesthesia (Y-axis). Each colored segment reflects a level of training exposure during anesthesiology specialization, ranging from “Once a year or less” to “Once a week or more”, as indicated in the legend. The X-axis shows the percentage of respondents in each current use category. The figure demonstrates a clear pattern: more frequent exposure to SADs during residency is associated with greater frequency of use in current practice.

Figure 2 shows the association between training exposure and current use: clinicians who used SADs regularly during residency were more likely to report frequent use in current practice. This was supported by Spearman correlation (ρ = 0.60, p < 0.001), indicating a moderate-to-strong positive relationship.

SADs were generally perceived as safe: 76.9% of respondents rated them as “almost always” or “always” safe, and 74.3% considered ventilation via SADs “almost always” or “always” adequate (Table 3). The lack of operator experience was the most commonly cited barrier, with 81.5% agreeing it limited broader use. Only 18.3% felt fully confident distinguishing between first- and second-generation devices.

**Table 3:** Perceptions, barriers, and training exposure related to Supraglottic Airway Devices (SADs) use.

Variable		n	%
Degree of safety			
	Not safe at all	2	0.5
	Rarely safe	8	1.9
	Sometimes safe	90	20.8
	Usually, safe	235	54.4
	Always safe	97	22.5
Adequate ventilation			
	Not effective at all	3	0.7
	Rarely effective	7	1.6
	Sometimes effective	101	23.4
	Usually effective	292	67.6
	Always effective	29	6.7
Supposed barriers to use			
Cost	Strongly disagree	56	13.0
	Disagree	131	30.3
	Neither agree nor disagree	83	19.2
	Agree	139	32.2
	Totally agree	23	5.3
Availability	Strongly disagree	38	8.8
	Disagree	95	22.0
	Neither agree nor disagree	59	13.7
	Agree	186	43.1
	Totally agree	54	12.5
Experience of professionals	Strongly disagree	7	1.6
	Disagree	40	9.3
	Neither agree nor disagree	33	7.6
	Agree	255	59.0
	Totally agree	97	22.5
Knowledge of the differences between 1^st^ and 2^nd^ generation SADs			
	No knowledge at all	10	2.3
	Basic understanding	111	25.7
	Moderate understanding	123	28.5
	Good understanding	109	25.2
	Excellent understanding	79	18.3
Elective surgeries in which you would use a SAD			
	Videolaparoscopic gastrointestinal surgery	29	6.7
	Videolaparoscopic urogenital surgery	94	21.7
	Open gastrointestinal tract	62	14.3
	Open urogenital	233	53.8
	Upper limbs	369	85.2
	Lower limbs	366	84.5
	Head and neck	35	8.1
	Thoracoabdominal wall	247	57.0
	Ophthalmology	262	60.5
	ENT (except oropharynx and larynx)	101	23.3
	Spine	7	1.6
	Cardiac	3	0.7
	Thoracic	6	1.4
	Caesarean section	29	6.7
	I wouldn't use it in any situation	12	2.8
Potential limitations to use			
	Regurgitation	298	68.8
	Aspiration of gastric contents	297	68.6
	Failed 1st attempt	29	6.7
	Insertion failure	195	45.0
	Low sealing pressure	156	36.0
	Inadequate ventilation	314	72.5
	Sore throat	51	11.8
	Hoarseness	23	5.3
	Post-operative nausea and vomiting	12	2.8
	Coughing on waking	7	1.6
	Dysphagia	39	9.0
	Tissue damage	61	14.1
	Insertion time	122	28.2
	No limitations	10	2.3
Access to information would increase the use of SADs			
	Strongly disagree	4	0.9
	Disagree	7	1.6
	Neither agree nor disagree	26	6.0
	Agree	164	38.0
	Totally agree	231	53.5

SAD, Supraglottic Airway Device.

SADs were most commonly selected for upper (85.2%) and lower limb surgeries (84.5%), followed by ophthalmology and thoracoabdominal wall procedures. Their use was less favored for spinal, thoracic, and cardiac surgeries. The primary hypothetical concerns limiting their use were inadequate ventilation (72.5%), regurgitation (68.8%), and aspiration of gastric contents (68.6%).

A large majority (91.5%) of respondents agreed that greater access to training and information would likely encourage broader implementation of SADs in anesthetic practice.

Binomial logistic regression analysis revealed that self-reported experience with SADs (OR = 14.8; 95% CI 5.5‒38.0, p < 0.001), the frequency of use during anesthesiology specialization (OR = 7.3; 95% CI 3.8‒14.3, p < 0.001) and age ≥ 45 (OR = 2.0; 95% CI 1.0‒4.0, p = 0.035) were associated with a greater chance of using the devices in clinical practice. Knowledge of device generation and professional workplace (public or private hospital) were not associated with its use (p > 0.05).

## Discussion

This national survey likely provides the first comprehensive snapshot of SADs use, perceptions, and barriers among Brazilian anesthesiologists. While SADs are widely recognized as safe and effective, our findings reveal considerable variability in their use across regions. This variation appears to be shaped by multiple factors, including differences in training, clinical confidence, institutional norms, and access to equipment. These patterns carry implications not only for Brazil, but also for other middle-income countries and health systems with internal disparities.

Participants mostly viewed SADs as safe and effective for ventilation, in line with robust international evidence supporting their use in both elective and emergency settings.^4^^,^^5^^,^^15^, ^16^, ^17^, ^18^ Large-scale studies, randomized trials, and meta-analyses have shown that SADs, particularly second-generation ones, reduce perioperative complications and enhance recovery compared to tracheal intubation.^4^^,^^15^^,^^18^ However, real-world usage remains limited: only one-third of respondents reported weekly use, and fewer than one in five felt confident distinguishing between first- and second-generation devices.

These findings highlight a persistent gap in training and continuing education. Accordingly, early and structured exposure during residency was associated with current use, reinforcing the critical role of specialist training in shaping long-term practice. Similarly, lack of operator experience emerged as the most frequently cited barrier, underscoring the need to strengthen education through simulation, clinical mentorship, and structured airway curricula ‒ particularly in regions where SADs remain underused.

The educational gap is especially relevant in light of recent advances such as video-guided SADs, which aim to improve device placement, airway seal, and anatomical visualization.^2^^,^^19^ While promising, the successful adoption of such innovations depends on a foundational understanding of existing SADs technologies. Our findings suggest that even this baseline familiarity remains limited in many settings, particularly those with fewer resources. To avoid widening practice disparities, global stakeholders must prioritize basic training and knowledge dissemination alongside technological advancement.

In addition to training, limited access to SADs remains a key constraint.^20^ Nearly 30% of respondents reported only partial availability in their workplaces. This disconnect between perceived clinical value and real-world access highlights ongoing logistical and procurement barriers that must be addressed to enable broader and more equitable use in routine anesthesia care.

Regional differences further illustrate these challenges. In the South ‒ a more well-resourced region ‒ over 80% of respondents reported using SADs weekly. In contrast, in the North ‒ where healthcare infrastructure is often more limited ‒ only 15% reported weekly use, while nearly one-third used them just once per year or less. These findings reflect broader patterns of inequity and call for targeted interventions to improve access and training in underserved regions.^20^, ^21^, ^22^, ^23^

Such disparities are not unique to Brazil. Given its continental scale and internal diversity, Brazil represents a microcosm of global anesthetic practice ‒ with regions resembling high-income settings and others facing constraints more typical of low-resource environments. These findings therefore offer transferable insights into the barriers that affect SADs implementation in a variety of global contexts.^20^, ^21^, ^22^, ^23^

Our data also suggest a cautious approach to the types of surgical procedures in which SADs are used. While upper and lower limb surgeries were commonly cited as appropriate, SAD use in gastrointestinal, laparoscopic, and thoracic procedures remained limited. The most frequent concerns were inadequate ventilation, regurgitation, and aspiration ‒ despite strong evidence supporting the safety of second-generation devices, even in high-risk populations.^3^^,^^4^^,^^6^^,^^7^ Furthermore, the perception of an “appropriate surgical duration” for the use of supraglottic airway devices may represent a potential barrier to wider adoption. Although this aspect was not specifically explored in this study, it is an important dimension of clinical decision-making that may warrant further investigation in future research. This reinforces the need for targeted educational efforts to update clinical perceptions and expand the indications for SADs use.

Similar challenges have been reported internationally. In China ‒ a continental country with regional disparities similar to Brazil ‒ a national survey found wide variation in airway management strategies for difficult airways and emphasized training and education as priorities for standardization.^22^ In India, a national survey among anesthesiologists also revealed regional variability in both equipment access and airway management practices, further underscoring the need to improve training and ensure equitable availability of airway tools.^20^ Notably, even in high-income settings such as the United Kingdom, despite widespread access to airway tools, a survey revealed considerable variation in SADs use during in-hospital cardiac arrests, reinforcing the influence of institutional culture, education, and confidence on practice.^23^ An international survey across 54 countries likewise reported broad inconsistencies in laryngeal mask airway use, with variability in perceived contraindications, procedure types, and patient characteristics. The authors called for broader dissemination of evidence-based guidelines to harmonize practice and improve safety.^21^

This study has several limitations. First, the use of convenience sampling and voluntary participation through social media channels may introduce selection bias and limit generalizability. Second, the survey relied on self-reported data, which may be subject to recall bias or social desirability effects. Of note, while a cross-sectional survey can rapidly provide a contemporary snapshot of practice and help describe a complex scenario, it does not permit firm causal inferences and is prone to biases and unmeasured confounding. Third, the questionnaire was not formally validated as a psychometric instrument. Fourth, we did not independently verify the institutional availability of SADs. Fifth, we did not delve into whether the perception of the device quality correlates with the generation available (first versus second generation SADs) or with the frequency of use. These factors may influence the notions and preferences reported by physicians and could help contextualize some of the findings. Exploring these associations in subsequent studies may provide additional insights into practice patterns. Sixth, although the collected sample met the a priori estimated size and allowed for precise analyses, the overall response rate was modest (4.4% total, 3.3% included), raising the potential for nonresponse bias and the possibility that respondents may differ meaningfully from nonrespondents. Nevertheless, these rates are comparable to those reported in other large-scale, web-based surveys of healthcare professionals and reflect the well-documented challenges of engaging clinicians through electronic platforms.^20^^,^^21^ Finally, although the study captured a wide national distribution, representation from certain regions was limited. This may affect the extent to which the findings reflect regional practice variability. Even so, the inclusion of over 400 fully completed questionnaires provides valuable insight into national practice patterns and offers lessons that may be relevant to other settings with similar healthcare challenges.

## Conclusion

This survey reveals that although SADs are broadly recognized as safe and effective, their adoption remains constrained by disparities in training, education, and access. Persistent concerns about certain clinical scenarios may also contribute to underuse. These findings emphasize the need to expand educational opportunities, modernize training programs, and ensure equitable access to airway devices.

## Data availability statement

The datasets generated and/or analyzed during the current study are available from the corresponding author upon reasonable request.

## Authors’ contributions

Jayme Marques dos Santos Neto: Refined the questionnaire; Secured ethical approval; coordinated survey distribution with the Brazilian Society of Anesthesiology, organized the dataset and wrote the manuscript.

Flávia Jatobá de Barros: Refined the questionnaire; secured ethical approval; coordinated survey distribution with the Brazilian Society of Anesthesiology, and organized the dataset.

Victor Macedo Lemos: Refined the questionnaire; secured ethical approval; coordinated survey distribution with the Brazilian Society of Anesthesiology, and organized the dataset.

Clístenes Crístian de Carvalho: Conceived the study idea; proposed the survey-based design; drafted the initial version of the questionnaire; conducted statistical analyses and wrote the manuscript.

All authors critically reviewed the final draft and approved the submitted version.

## Conflicts of interest

The authors declare no conflicts of interest.
